# Corneal endothelial cells changes in different stages of Keratoconus: a multi-Centre clinical study

**DOI:** 10.1186/s12886-021-01913-7

**Published:** 2021-03-21

**Authors:** Ahmed Elmassry, Ahmed Osman, Moataz Sabry, Mohamed Elmassry, Mai Katkat, Mohamed Yousry Hatata, Mohamed El-Kateb

**Affiliations:** 1 Department of Ophthalmology, Faculty of Medicine, Alexandria University, Champollion Street, Al Attarin, Alexandria, Egypt; 2 Department of Ophthalmology, Tanta University, Tanta, Egypt; 3 Department of Ophthalmology, Port Said University, Port Fuad, Egypt; 4 Alex I-Care Hospital, Alexandria, Egypt

**Keywords:** Corneal endothelial cells, Keratoconus, Specular microscopy

## Abstract

**Purpose:**

To assess the corneal endothelial cells morphology and count in keratoconus patients and their correlation with different stages of keratoconus.

**Methods:**

Prospective non randomized multi-centric clinical study included 150 eyes of 150 keratoconus patients. Four centers in Egypt participated in this study included: Departments of Ophthalmology in Alexandria University, Tanta University and Port Said University and Alex I-Care hospital. Pentacam (Wavelight Oculyzer II) and specular microscopy (Tomey EM-3000) were done to all eyes. Keratoconic eyes were classified according to Amsler classification into stage 1, 2 and 3. Stage 1 included 99 eyes, stage 2 included 32 eyes & stage 3 included 19 eyes.

**Results:**

The mean age of keratoconus patients was 24.07 ± 6.154 years. Forty five cases were males (30%) and 105 cases were females (70%). There was statistically significant difference in endothelial cell density (*p* < 0.001) and coefficient of variation (*p* = 0.012) between different stages of keratoconus eyes. Regarding cell surface area, there was statistically significant difference in cell surface area between different stages of keratoconus eyes (*p* < 0.001). In addition, for cell morphology, there was statistically significant difference between different stages of keratoconus eyes (*p* < 0.001).

**Conclusions:**

Qualitative and quantitative structural changes were seen in endothelial cells of keratoconus eyes by using specular microscopy. For stages 1 and 2, keratoconus may not affect the corneal endothelim significantly. The endothelium in stage 3 shows significant changes regarding polymegathism and pleomorphism.

## Introduction

Keratoconus (KC) is a corneal disorder with a non-inflammatory nature. The reported prevalence is one in 2000 people globally and showed variability among studies [1–10]. Although KC has a world-wide distribution, it was reported more in specific groups such as South Asians, North Africans, and Eastern Mediterranean [11–13].

There are variable rates of the progression between individuals and severe stages of the disease are not supposed to occur for everyone. In 10–15% of patients, it may reach for severe stages with required transplantation to get functional vision [1–10].

Corneal endothelium in humans acts as a one layer of uniformed cells having a hexagonal shape and covers the posterior surface of the cornea. Its function is to stabilize the hydration of corneal and confirm the transparency. As endothelial cells are not usually reproducible, the surrounding cells will replace both space and activity of dead cells. Consequently, age and pathologies affects the total cells number, regular tessellation, and their size [14].

The cornea health state can be described through analyzing morphometric parameters for the endothelium of the cornea which gives clinical relevant data. Likewise, endothelial density of cell, polymegethism (or variation coefficient, cell size differences expressed as fractional standard deviation of cell areas), together with pleomorphism (or hexagonality coefficient, hexagonal cells fraction over the total cells number) are parameters commonly used to characterize the condition of the endothelial cells quantitatively [15]. Confocal microscopy and in-vivo specular allow getting non-invasive images for the corneal endothelial layer of humans, from which morphometric parameters and density can be derived [16].

DALK represents a successful transplantation form with healthy endothelium [17–20]. When abnormalities in corneal endothelium are present in KC patients, these may theoretically affect the maintenance of the clarity for corneal graft in the long-term after DALK. When we correlate the grade of the disease with the extent of endothelial abnormality, this may change the criteria for selecting DALK. Therefore, in advanced abnormal endothelium, PK along with donor tissue of high-quality may be a better choice compared to DALK. Confocal microscopy and specular microscopy [21].studies have shown abnormal endothelium in patients with KC. The aim of this study was to assess the corneal endothelial cells morphology and count in KC patients and their correlation with different stages of KC.

### Patients and method

This prospective non comparative multi-centric clinical study included 150 eyes of 150 keratoconus patients. Four centers in Egypt participated in this study included: Departments of Ophthalmology in Alexandria University, Tanta University and Port Said University and Alex I-Care center between January 2019 and February 2020. The protocol of this study was accepted and approved by the Ethics Committee of Faculty of medicine, Alexandria University, Egypt on January 17th, 2019 with IRB No. 00012098, FWA No. 00018699 and Serial No. 0304218.

Keratoconic eyes were classified into 4 stages according to the classification of Amsler-Krumeich:

#### Stage 1

Eccentric bulging of the cornea, myopia and/ or astigmatism less than 5 D, corneal radius less than or equal to 48 D, no corneal opacities and Vogt’s striae.

#### Stage 2

Myopia and/ or astigmatism more than 5 D and less than 8 D, corneal radius less than or equal to 53 D, no central opacities and pachymetry at least 400 um.

#### Stage 3

Myopia and or astigmatism more than 8 D and less than 10 D, corneal radius more than 53 D, no central opacities and pachymetry 200–400 um.

#### Stage 4

Refraction difficult to determine, the radius of corneal more than 55 D, central scars and pachymetry less than 200 um.

Exclusion criteria included eyes with history of contact lens wearing, previous ocular surgeries, acute hydrops, previous collagen crosslinking and eyes with stage 4 keratoconus because of the corneal opacities that will interfere with accurate data captured by specular microscopy. There were no patients with forme fruste keratoconus (unilateral KC) included in our study.

Informed consents were obtained from all the participants in our study. Every patient was subjected to full ophthalmological examination included uncorrected and corrected distance visual acuity, examination by slit-lamp, corneal topography and thickness data from Scheimpflug camera (Oculyzer II, Wavelight Inc.). Corneal endothelial cells data where captured from the cornea and centered over the cone using non contact specular microscopy (Tomey EM-3000). The data obtained from the specular microscopy were the following:

***Cell Density (CD):*** is defined as the density of the analyzed endothelial cells as number of cells per 1 mm.

***Coefficient of Variation (CV):*** of the analyzed endothelial cells, derived by dividing standard deviation by the average dimension.

***Polymegathism:*** is defined as the difference in sizes and distribution of endothelial cells dimensions. From the output of polymegathism data, 200–300 um^2^ surface area cells percentage, 300–400 um^2^ surface area cells percentage and 400–500 um^2^ surface area cells percentage were selected.

***Plemorphism:*** is defined as the difference and distribution of endothelial cells shapes. From the output of plemorphism data, pentagonal, hexagonal and heptagonal cell morphology were be selected.

### Statistical analysis

Data were collected and analyzed using SPSS program for statistical analysis version 25. Kolmogorov-Smirnov test was used to check the normal distribution and when showed no significance, we used parametric statistics. Otherwise, the non-parametric statistics was used.

Data were described using mean, standard deviation, range for the normally distributed data and median and inter-quartile range for non-normally distributed data. Categorical data were described as frequency and percentage of total. For more than two groups, comparisons, One-way Analysis of Variance (ANOVA) test was used for normally distributed data with post-hoc multiple comparisons when ANOVA test was significant. Kruskal-Wallis (KW) test was used for non-normally distributed data with post-hoc pair-wise comparisons when KW is significant using Dunn-Bonferroni test for multiple comparisons. We adopted a randomly selected eye per a case to avoid inter-eye correlation [22, 23].

## Results

### Age and sex

The mean age of keratoconus patients was 24.07 ± 6.154 with range from 16 to 45 years. As regards the sex distribution, 45 cases were males (30%) and 105 cases were females (70%).

### Endothelial cell density (ECD) (cells/mm^2^)

Table 1 shows comparison between different stages of keratoconus and endothelial cell density (ECD). There was statistically significant difference in endothelial cell density between different stages of keratoconus eyes (*p* < 0.001).

**Table 1:** Comparison between different stages of keratoconus eyes according to the endothelial cell density and coefficient for variation

	Stage 1 (***n*** = 99)	Stage 2 (***n*** = 32)	Stage 3 (***n*** = 19)
**Endothelial cell density (ECD)**			
Range	2193–3434	2143–2971	1782–2654
Mean ± S.D	2734.25 ± 284.25	2453.25 ± 274.41	2344.05 ± 396.14
*P* value	< 0.001		
P1	< 0.001		
P2	< 0.001		
P3	0.625		
**Coefficient of Variation (CV)**			
Range	27–83	34–56	31–44
Mean ± S.D	38.45 ± 12.35	44.84 ± 6.02	38.32 ± 5.28
*P* value	0.012		
P1	0.011		
P2	1		
P3	0.106		

*P1 comparison between stage 1 and stage 2*

*P2 comparison between stage 1 and stage 3*

*P3 comparison between stage 2 and stage 3*

### Coefficient of variation (CV) (%)

Table 1 shows comparison between different stages of keratoconus and coefficient of variation (CV). There was statistically significant difference in coefficient of variation between different stages of keratoconus eyes (*p* = 0.012).

### Cell surface area 200–300 um^2^ (%)

Table 2 shows comparison between different stages of keratoconus and cell surface area 200–300 um^2^ (%). There was statistically significant difference in cell surface area 200–300 um^2^ between different stages of keratoconus eyes (*p* < 0.001).

**Table 2:** Comparison between different stages of keratoconus eyes according to cell surface area

	Stage 1 (n = 99)	Stage 2 (n = 32)	Stage 3 (n = 19)
**Cell surface area (200–300 um**^**2**^**)**			
Range	17–42	17–31	10–24
Mean ± S.D	26.91 ± 7.46	21.09 ± 5.27	17.37 ± 5.73
*P* value	< 0.001		
P1	< 0.001		
P2	< 0.001		
P3	0.188		
**Cell surface area (300–400 um**^**2**^**)**			
Range	1–1.2	1–1.2	1–1.2
Mean ± S.D	29.04 ± 3.81	24 ± 2.3	21.47 ± 4.54
*P* value	< 0.001		
P1	< 0.001		
P2	< 0.001		
P3	0.054		
**Cell surface area (400–500 um**^**2**^**)**			
Range	12–30	11–29	19–26
Mean ± S.D	20.62 ± 4.72	20.5 ± 5.12	24.68 ± 1.6
*P* value	0.002		
P1	1		
P2	0.001		
P3	0.005		

*P1 comparison between stage 1 and stage 2*

*P2 comparison between stage 1 and stage 3*

*P3 comparison between stage 2 and stage 3*

### Cell surface area 300–400 um^2^ (%)

Table 2 shows comparison between different stages of keratoconus and cell surface area 300–400 um^2^ (%). There was statistically significant difference in cell surface area 300–400 um^2^ between different stages of keratoconus eyes (*p* < 0.001).

### Cell surface area 400–500 um^2^ (%)

Table 2 shows comparison between different stages of keratoconus and cell surface area 400–500 um^2^ (%). There was statistically significant difference in cell surface area 400–500 um^2^ between different stages of keratoconus eyes (*p* = 0.002).

### Pentagonal cell morphology (%)

Table 3 shows comparison between different stages of keratoconus and pentagonal cell morphology (%). There was statistically significant difference in pentagonal cell morphology between different stages of keratoconus eyes (*p* < 0.001).

**Table 3:** Comparison between different stages of keratoconus eyes according to cell morphology

	Stage 1 (n = 99)	Stage 2 (n = 32)	Stage 3 (n = 19)
**Pentagonal cell morphology**			
Range	14–27	16–24	19–27
Mean ± S.D	19.72 ± 3.16	20.69 ± 3.23	24.37 ± 2.43
*P* value	< 0.001		
P1	0.378		
P2	< 0.001		
P3	< 0.001		
**Hexagonal cell morphology**			
Range	30–61	33–48	28–40
Mean ± S.D	48.74 ± 8.61	40.03 ± 5.29	32.42 ± 5.29
*P* value	< 0.001		
P1	< 0.001		
P2	< 0.001		
P3	0.002		
**Heptagonal cell morphology**			
Range	17–26	14–28	17–26
Mean ± S.D	20.75 ± 2.55	22.28 ± 4.71	22.68 ± 4.02
*P* value	0.014		
P1	0.074		
P2	0.064		
P3	1		

*P1 comparison between stage 1 and stage 2*

*P2 comparison between stage 1 and stage 3*

*P3 comparison between stage 2 and stage 3*

### Hexagonal cell morphology (%)

Table 3 shows comparison between different stages of keratoconus and hexagonal cell morphology (%). There was statistically significant difference in hexagonal cell morphology between different stages of keratoconus eyes (*P* < 0.001).

### Heptagonal cell morphology (%)

Table 3 shows comparison between different stages of keratoconus and heptagonal cell morphology (%). There was statistically significant difference in heptagonal cell morphology between different stages of keratoconus eyes (*p* = 0.014).

## Discussion

In the present study, we discussed the relation between different stages of keratoconus and endothelial cells changes as regarding the endothelial cell density, coefficient for variation (CV), polymegathism and pleomorphism.

Our study included 150 eyes of 150 KC patients. The KC eyes were classified into 4 stages according to Amsler’s classification. Stage 4 eyes were not included in the study because of the permanent scarring that interferes with specular microscopic images.

Since keratoconus is an ectatic disease affecting both the anterior and posterior corneal surfaces, there might be changes in corneal endothelial cell number and morphology, especially in advanced stages of the disease. In keratoconus, evaluation of the corneal endothelium may be important since theoretically these cells may be damaged as a result of microscopic ruptures in Descemet’s membrane in ectatic areas, ultraviolet radiation damage due to stromal thinning, chronic eye rubbing, long-term contact lens wear, and oxidative stress [22].

### Endothelial cell density (ECD)

In our study the analysis of ECD in different stages of keratoconus revealed significant difference. Only 5 studies compared ECD in different stages of KC in the literature. Timucin et al. [24], Niederer et al. [25] and El-Agha et al. [26] found no significant difference in ECD between mild, moderate and severe stages of KC. While Uçakhan et al. [27] and Bitirgen G [28] found significant reduction in ECD in severe stages when compared with mild and moderate stages of keratoconus.

### Coefficient of variation (CV) and Polymegathism

In our study, there were statistically significant differences between coefficient of variation (CV) and different stages of keratoconus. El-Agha et al. [26] found that CV to be from 22 to 67% and was higher in stage 3. However, this difference was not of statistical significance (*p* = 0.51).

Hollingsworth et al. [29] found that the level of polymegethism in endothelium present in the KC eye was found to be as for matched controls (*p* = 0.08). Their results are consistent with the findings of Halibis [30]. He showed that the level of polymegethism in KC subjects is not different compared to of lens-wearing subjects. None of the previous studies or others have however tried to correlate the degree of polymegathism in endothelium with the stage of KC.

In our study we analysed the polymegathism results according to cell surface area of the endothelial cells in keratoconic eyes. Our results revealed significant difference between eyes with different stages of keratoconus.

### Pleomorphism

The hexagon is the polygon with the greatest surface area in relation to its perimeter. Hence, it is an efficient cell shape for covering a given area. In normal cornea, 60% of the endothelial cells are expected to be hexagons. Decrease to the normal distribution of 60% for 6 sided cells to a lesser percentage can be the result of stress to the endothelial cells.

Our results revealed significant difference between eyes with pleomorphism and different stages of keratoconus. Conversely, Laing et al. [21] used specular microscopy to study the corneal endothelium in 12 eyes with KC. The finding showed an increase for the pleomorphism of cells with some of the cells smaller compared to normal and considerably distributed through the endothelial cell population. Likewise, Matsuda et al. [21] found that hexagonal cells in keratoconus were significantly lower than that of controls and also there was a significant increase in the pleomorphism of cells. The mean endothelial hexagonality percentage in a study by Uçakhan et al. [27] by confocal microscopy was statistically significantly lower in KC eyes than in control eyes (*P* < 0.05) and was lower in severe KC but this difference was not of clinical significanc. El-Agha et al. [26] found that the percentage for hexagonal cells can range from 38 to 78 and it may be higher in stage 1 compared to in stages 2 and 3. However, this difference was not significant (*p* = 0.51). Comparing mild-to-moderate KC (stages 1 and 2) with severe KC (stage 3), the difference was also not of statistical significance (*p* = 0.4).

## Conclusions

From this study, we concluded that qualitative and quantitative structural changes were observed in endothelial cells of eyes with KC. In stages one and two, KC does not significantly affect the corneal endothelim. The endothelium in stage 3 shows significant changes regarding polymegathism and pleomorphism. Our study can recommend that penetrating keratoplasty maybe superior to DALK in the management of stage 3 keratoconus as the endothelial cells changes will interfere with the stability of DALK. This recommendation needs further studies with long durations of follow up.

## Data Availability

The data used to support the findings of this study are available from the corresponding author upon request.

## Abbreviations

KC: Keratoconus
CD: Cell Density
CV: Coefficient of Variation
ANOVA: One-way Analysis of Variance
ECD: Endothelial cell density
